# Advances in multi-dimensional coherent spectroscopy of semiconductor nanostructures

**DOI:** 10.1080/23746149.2017.1346482

**Published:** 2017-07-17

**Authors:** Galan Moody, Steven T. Cundiff

**Affiliations:** a Applied Physics Division, National Institute of Standards & Technology, Boulder, CO, USA; b Department of Physics, University of Michigan, Ann Arbor, MI, USA

**Keywords:** Multi-dimensional coherent spectroscopy, semiconductors, nanostructures, excitons, coherence, 78.47.jh Ultrafast spectroscopy, 78.47. nj Nonlinear optical spectroscopy, 78.67.-n Optical properties of nanostructures, 71.35.-y Excitons

## Abstract

Multi-dimensional coherent spectroscopy (MDCS) has become an extremely versatile and sensitive technique for elucidating the structure, composition, and dynamics of condensed matter, atomic, and molecular systems. The appeal of MDCS lies in its ability to resolve both individual-emitter and ensemble-averaged dynamics of optically created excitations in disordered systems. When applied to semiconductors, MDCS enables unambiguous separation of homogeneous and inhomogeneous contributions to the optical linewidth, pinpoints the nature of coupling between resonances, and reveals signatures of many-body interactions. In this review, we discuss the implementation of MDCS to measure the nonlinear optical response of excitonic transitions in semiconductor nanostructures. Capabilities of the technique are illustrated with recent experimental studies that advance our understanding of optical decoherence and dissipation, energy transfer, and many-body phenomena in quantum dots and quantum wells, semiconductor microcavities, layered semiconductors, and photovoltaic materials.

## Graphical abstract

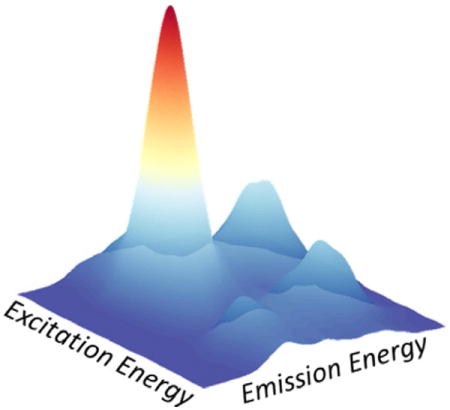

## 1. Introduction

A central challenge in condensed-matter, atomic, and molecular science is to understand the roles that numerous physical processes responsible for decoherence and relaxation play in the complexity of light-matter interaction. Condensed matter comprises ~10^23^ particles cm^−3^ that interact through the infinite-range Coulomb force, which is responsible for the formation of excitons (bound electron-hole pairs) and residual interactions between them. In semiconductors, the optical response near the fundamental bandgap is profoundly influenced by Coulomb correlations and other many-body effects associated with excitons, in marked contrast to atomic systems [1,2]. Substantial physical insight into the implications of many-body phenomena on exciton dynamics has been acquired through time-resolved spectroscopy [3,4]. An effective technique must have the dynamic range to probe the optical response across femtosecond to nanosecond timescales and the sensitivity to resolve disorder due to stochastic fluctuations in structural and material composition ubiquitous to semiconductor nanostructures. One-dimensional (1D) optical spectroscopies have the requisite timing resolution, but a 1D spectrum provides an ensemble-averaged response that can be challenging to interpret, especially for heterogeneous systems [5]. In semiconductors, random disorder manifests as a distribution of transition frequencies that inhomogeneously broadens the optical lines. Resolving the intrinsic homogeneous linewidth of each individual emitter in an ensemble is generally impossible with 1D spectroscopy. Although 1D spectra may contain signatures of couplings between transitions, insight into their origin and dynamics is usually obscured by multiple overlapping contributions in the optical response.

Multi-dimensional coherent nonlinear spectroscopy (MDCS) techniques have been developed to address these challenges [6]. MDCS relies on the interaction of a sequence of ultrafast optical pulses with a nonlinear medium to produce a transient four-wave mixing (FWM) signal that contains all the details of the χ^(3)^ optical response. By correlating the phase evolution of the FWM signal across two (2DCS) or three (3DCS) time periods and taking a multi-dimensional Fourier transform, the optical response is unraveled onto multiple frequency axes. Multi-dimensional coherent spectra provide a more comprehensive picture of the dynamical evolution of excitons in semiconductors compared to 1D coherent spectra, which are projections of a multi-dimensional spectrum onto a single frequency axis. Compared with conventional 1D spectroscopies, advantages of MDCS include: (i) simultaneous measurement of the homogeneous and inhomogeneous optical linewidths in heterogeneous ensembles; (ii) unambiguous identification of different types of couplings and interactions between resonances; (iii) separation of different quantum pathways contributing to the nonlinear optical response; and (iv) full characterization of the amplitude *and phase* of the nonlinear response, which is sensitive to many-body effects.

In this article, we review recent advances in MDCS experiments of semiconductor nanostructures. We present fundamental aspects of light-matter interaction in semiconductors using the density matrix formalism and double-sided Feynman diagrams [7]. Different experimental implementations of MDCS and the theoretical basis for extracting quantitative details of exciton interactions and optical linewidths are discussed. We begin the review of experiments with 2DCS of semiconductor quantum dots (QDs), which form at the interface of a thin quantum well (QW). Zero-dimensional QDs are a model system for demonstrating how 2DCS enables unambiguous separation of homogeneous and inhomogeneous contributions to the optical linewidth of an ensemble [8,9]. The ability of 2DCS to measure different populations within the ensemble also allows it to serve as a sensitive probe of Rabi oscillations by independently tracking disparate QD populations with different Rabi frequencies [10]. Hyperspectral imaging of the FWM signal elucidates coherent coupling between excitons in isolated QDs [11].

In higher-dimensional systems, such as an asymmetric double QW, an analysis of resonance lineshapes, frequencies, and amplitudes from multiple types of 2D and 3D spectra reveal the role of many-body effects in coupling and energy transfer between excitons confined to separate wells [12,13]. Strong light-matter coupling is also possible by embedding a QW into a semiconductor microcavity, which leads to hybridized exciton-polariton states; two-, three-, and four-quantum 2D spectra are particularly sensitive to interactions between different polariton branches [14–16]. As the dimensionality of the QW is reduced to an atomic thickness, such as in monolayer van der Waals semiconductors, reduced dielectric screening enhances Coulomb interactions and correlations between electrons. In 2D spectroscopy, strong many-body effects manifest as isolated peaks associated with neutral excitons, charged excitons, and bound biexcitons with an order-of-magnitude larger binding energy compared to conventional semiconductors with higher dimensionality [17–19]. Pronounced exciton-exciton interactions lead to rapid dephasing at high exciton densities [20,21], and exciton-phonon scattering enhances energy transfer in 2D monolayers as well as 1D carbon nanotubes [22]. Energy transfer and charge transport also play an important role in photovoltaics, where multi-exciton generation in PbS photocells produces distinct dispersive features in 2D photocurrent spectroscopy that are difficult to detect with other methods [23].

## 2. Multi-dimensional coherent spectroscopy of semiconductor nanostructures

The Fourier-transform methods of MDCS were originally developed at radio frequencies for nuclear magnetic resonance spectroscopy of spins [24]. Over the last two decades, much work has been devoted to implementing these concepts in the optical regime. In the infrared, MDCS provides a detailed view of vibronic coherences that elucidates the structure and dynamics of molecules [25–27]. At visible optical frequencies, MDCS has becoming an increasingly useful tool for probing electronic transitions, with significant contributions in identifying long-lived coherences in light-harvesting photosynthetic complexes [28–33], electronic-vibrational coupling in nitrogen-vacancy centers [34], collective resonances in atomic vapors [35,36], and electronic structure in colloidal nanocrystals [37–40]. The limits of MDCS have recently been extended to the extreme ultraviolet spectral region to explore inner-valence transitions in complex molecules [41,42]. In this article, recent experimental developments in the application of MDCS to study excitonic dynamics, interactions, and transport in semiconductor nanostructures are reviewed.

### 2.1. Optical properties of semiconductors

**Figure 1:**
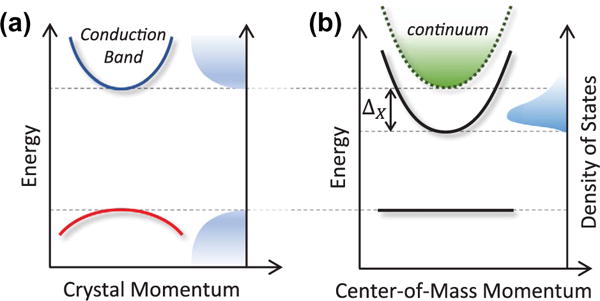
(a) Electronic band diagram of a direct bandgap semiconductor, where the conduction band minimum and valence band maximum are aligned in crystal momentum space. (b) In the excitation picture, optically excited electron-hole pairs form bound hydrogenic states called excitons with zero center-of-mass momentum and a binding energy Δ_X_ relative to the continuum of unbound, free electron-hole pairs. The density of states is concentrated near the exciton resonance.

Semiconductors are generally classified as having either a direct or indirect bandgap in their energy-momentum dispersion. Direct gap semiconductors absorb and emit light efficiently, since the minimum of the conduction band and the maximum of the valence band reside at the same point in the crystal momentum *k*-space (see Figure 1(a)). The canonical direct gap semiconductor for coherent spectroscopy experiments is gallium arsenide (GaAs) and its compounds, which comprise materials from groups *III* and *V* of the periodic table. The fundamental bandgap of GaAs appears at the Γ-point in *k*-space and consists of a single conduction band with effective mass *m_c_* = 0.0662*m_e_*, a heavy-hole valence band with *m_hh_* = 0.34*m_e_*, and a light-hole valence band with *m_lh_* = 0.094*m_e_* (only one valence band is shown in Figure 1 for simplicity) [43]. The most widely studied semiconductor nanostructures are based on *III*–*V* compound heterostructures, including ternary alloys such as InGaAs and AlGaAs, owing to their amenable optical properties for efficient light-matter interaction, ability to be grown in stacked layers with atomic precision using molecular beam epitaxy, and compatibility with nanofabrication processing techniques.

Because optically excited semiconductors are inherently a many-body system, Coulomb interactions between electrons and holes give rise to pronounced excitonic effects that dominate the band-edge optical response. At cryogenic temperatures, heavy-hole and light-hole excitons appear as sharp peaks in the linear optical spectrum near ~1.515 eV, which is below the direct bandgap of GaAs at 1.519 eV by the exciton binding energy Δ_X_ = 4.1 meV (see Figure 1(b)). In bulk GaAs, the valence bands are degenerate at *k* = 0; however, quantum confinement and mechanical strain lift the degeneracy, typically leading to 5–10 meV splitting between heavy-hole and light-hole exciton states. A prototypical example of a semiconductor nanostructure is a quantum well (QW) formed by sandwiching a thin GaAs layer between two layers of Al_x_Ga_1–x_As. A typical alloy ratio of *x* = 0.3 results in a 245 meV deep QW in the conduction band and 130 meV for the valence band. For a sufficiently thin GaAs layer (e.g. 10 nm), quasi-2D quantum confinement of electrons and holes increases the exciton binding to Δ_X_ ≈ 10 meV.

An important component of semiconductor QWs is the interface between the well and the barrier materials. Due to the stochastic nature of the epitaxial growth process, monolayer well-width fluctuations introduce interfacial surface roughness that acts as shallow in-plane disorder potentials for excitons. Well-width fluctuations lead to an inhomogeneous distribution of exciton frequencies that broadens the optical linewidth. For sufficiently narrow QWs in which the fluctuations represent a significant percentage of the well width, excitons can become localized at the fluctuation site, which acts as a zero-dimensional quantum confinement potential known as a ‘natural’ or interfacial QD with typical confinement energy of ~10 meV relative to free excitons in the QW [44]. ‘Self-assembled’ islands can also form in narrow InAs/GaAs QWs due to strain from the large lattice mismatch between indium and gallium. Strong confinement in self-assembled QDs is more reminiscent of an atomic two-level system (see Section 3).

The basic concepts of excitons in GaAs heterostructures also applies to other semiconductors if the exciton Bohr radius extends over many lattice sites (Wannier-Mott-type), which is true for the systems reviewed in this article. Although historically *III*–*V* semiconductor heterostructures have been the material-of-choice for coherent spectroscopy experiments, recent advances in material growth, processing, and semiconductor fabrication technologies have afforded new opportunities for exploring coherent light-matter interaction in more complex heterostructures. A natural extension of a single QW is the asymmetric double QW (DQW) in which electron and hole wavefunctions become delocalized across both wells separated by a narrow barrier. Experiments on InGaAs/GaAs DQWs reveal unexpected interactions associated with many-body effects even in quantum-mechanically isolated QWs (Sections 4 and 5).

When confined to a truly 2D system, interesting exciton physics emerges. Two-dimensional (2D) excitons have only recently been realized in monolayer transition metal dichalcogenides (TMDs), which are atomically thin semiconductors with a direct gap at the *K* point in *k*-space. The heavier electron and hole effective masses in TMDs (both on the order of 0.5*m_e_*) compared to *III*–*V* materials, in combination with reduced dielectric screening of the Coulomb interaction in two dimensions, leads to an enhancement of the exciton binding energy to 100’s of meV, making them stable even at room temperature. Two-dimensional materials and their heterostructures offer a new avenue for controlling the optical and electronic response, which has been leveraged to tune the exciton energy and charge transport properties in single-walled carbon nanotubes (Section 4).

Interest in controlling the optical properties of semiconductors extends to other types of nanostructures, including semiconductor quantum wells embedded in a microcavity and colloidal nanocrystals grown in solution. While the former provides an excellent test of many-body physics in the regime of strong light-matter interaction (Section 6), the latter finds practical applications for photodetection, photoemission, and photovoltaics (Section 7).

### 2.2. Coherent light-matter interaction

**Figure 2:**
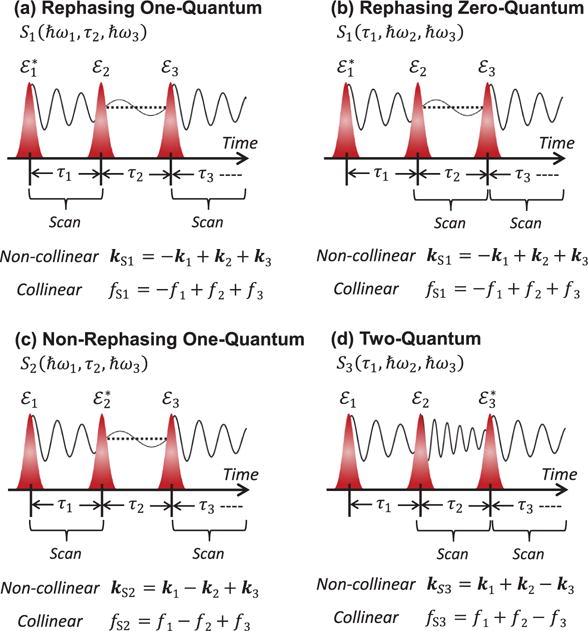
Pulse time-ordering sequences for MDCS experiments. Rephasing, or ‘photon echo’, (a) one- and (b) zero-quantum pulse sequence in which the conjugate field $\left(\varepsilon_{1}^{*}\right)$ arrives at the sample first. (c) Non-rephasing one-quantum pulse sequence in which the conjugate field arrives second. (d) Two-quantum pulse sequence in which the conjugate field arrives third. Separation of the four-wave mixing signal from other linear and nonlinear contributions to the polarization is possible through phase-matching or dynamic phase-cycling techniques for non-collinear or collinear pulse propagation geometries, respectively.

MDCS techniques rely on sufficiently strong optical fields to drive the system beyond the linear regime, i.e. higher order contributions to the induced polarization in the material are relevant and can be expressed as

(1) $$P=\chi^{\left(1\right)}\varepsilon +\chi^{\left(2\right)}\varepsilon^{2}+\chi^{\left(3\right)}\varepsilon^{3}+\ldots$$

where *P* is the induced polarization (omitting the vector and tensor notation), χ^(n)^ is the *n*^(th)^-order susceptibility, and *ε* is the incident electric field. In semiconductors with spatial inversion symmetry, the lowest-order nonlinearity stems from the χ^(3)^ susceptibility. χ^(3)^ processes give rise to several optical phenomena including self-phase modulation, Kerr-lens mode-locking, and four-wave mixing (FWM), which is at the heart of MDCS methods. In FWM experiments, the output from a mode-locked oscillator or parametric amplifier is divided into a series of four phase-coherent replicas using optical interferometers or diffractive optics. The field of each pulse can be expressed as $\varepsilon_{j}(r,t)={\hat{\varepsilon }}_{j}e^{i(k_{j}r-\omega_{j}t)}+c.c.$, where ${\hat{\varepsilon }}_{i}$, **k***_i_*, and *ω_i_*, and are the field envelope, wavevector, and angular frequency of the *j*th pulse. The total field entering the expression for the third-order polarization, *P*^(3)^ = *χ*^(3)^*ε*^3^, is a sum of the three incident pulses: $\varepsilon (r,t)=\sum_{j}\varepsilon_{j}\left(r_{j},\omega_{j},t-t_{j}\right)$, where the *j*th pulse arrives at time *t_j_*. In a three-pulse FWM experiment, only terms that depend on various permutations of the product $\varepsilon_{1}^{*}\varepsilon_{2}\varepsilon_{3}$ contribute to the third-order polarization,

(2) $$P^{\left(3\right)}(r,t_{1},t_{2},t_{3})=\int_{0}^{\infty }{ℛ}^{\left(3\right)}\left(t_{1}^{\prime },t_{2}^{\prime },t_{3}^{\prime }\right)\varepsilon_{1}\left(r,\;t_{1}^{\prime }-t_{1}\right)\varepsilon_{2}\left(r,\;t_{2}^{\prime }-t_{2}\right)\varepsilon_{3}\left(r,\;t_{3}^{\prime }-t_{3}\right){\text{dt}}_{1}^{\prime }{\text{dt}}_{2}^{\prime }{\text{dt}}_{3}^{\prime },$$

where ${ℛ}^{\left(3\right)}$ is the third-order response function that contains details of the coherent light-matter interaction, including the material’s optical dipole moments, transition frequencies, excited-state dynamics, etc. Pulse sequences are shown in Figure 2 for various time-orderings, which are distinguished by the arrival time of the conjugated field denoted as $\varepsilon_{j}^{*}$. Varying the time-ordering of the pulses provides access to different quantum mechanical pathways connecting various excited states in the system [7,45], as described in more detail in Section 2.3. The FWM signal field can be isolated from other contributions to the polarization through spatial filtering or dynamic phase-cycling algorithms for non-collinear or collinear pulse propagation geometries, respectively. These methods are discussed in more detail in Section 2.4.

**Figure 3:**
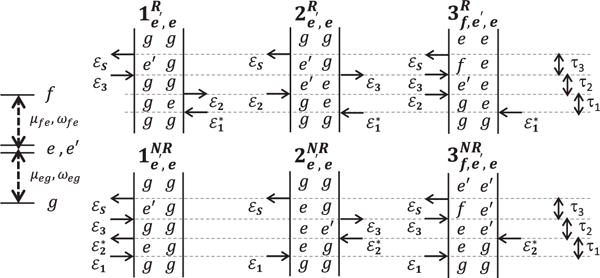
Quantum pathways for a three-level ladder system represented by double-sided Feynman diagrams in the rephasing (top row) and non-rephasing (bottom row) pulse time ordering. Diagrams 1, 2, and 3 correspond to ground-state bleaching (GSB), excited-state emission (ESE), and excited-state absorption (ESA) quantum pathways, respectively.

The density matrix formulism provides a convenient framework for generating sum-over-states expressions for ${ℛ}^{\left(3\right)}$. In the dipole approximation, the equations of motion for the matrix elements of the density operator *ρ*, known as the optical Bloch equations, are

(3) $${\dot{\rho }}_{ij}=\frac{-i}{\hbar }\sum_{k}\left(H_{ik}\rho_{kj}-\rho_{ik}H_{kj}\right)-\gamma_{ij}\rho_{ij},$$

where relaxation described by *γ_ij_* has been added phenomenologically. In Equation (3), *H* is the total system Hamiltonian with the light-matter interaction given by –***μ*** · *ε*(***r***, *t*), the relaxation rate $\gamma_{ij}=\left(\Gamma_{i}+\Gamma_{j}\right)/2+\gamma_{ij}^{ph},\Gamma_{i}\left(\Gamma_{j}\right)$ is the decay rate of state *i* (*j*), and $\gamma_{ij}^{ph}(i\neq j)$ is the elastic dephasing rate corresponding to decoherence from scattering that does not affect the population state. FWM experiments are performed in the weak-field limit, where the light-matter interaction can be treated perturbatively. Expanding the optical Bloch equations in orders of the Rabi frequency $\Omega \equiv \mu \hat{\varepsilon }/\hbar$, we arrive at the *n*th order expression (in the rotating-wave approximation)

(4) $${\dot{\rho }}_{ij}^{\left(n\right)}=-i\Delta_{ij}\rho_{ij}^{\left(n\right)}+\frac{i\Omega }{2}\rho_{kl}^{\left(n-1\right)},$$

where Δ*_ij_* ≡ *ω_i_* – *ω_j_* − *iγ_ij_* and *n* is the order of perturbation. The interaction of each optical field with the system increases the order of perturbation from *n−1* to *n*. An examination of Equation (4) reveals that odd-orders of the light-matter interaction generate superpositions, or coherences, between states (off-diagonal elements of *ρ*) and even-orders generate populations (diagonal elements). A multi-dimensional spectrum can be modeled by summing expressions from Equation (4) up to third-order for all quantum pathways connecting states *i* and *j* and then taking a multi-dimensional Fourier transform. Each quantum pathway can be conveniently represented by a double-sided Feynman diagram, which provides an intuitive conceptualization of the contributions to the nonlinear optical response. As an example, generalized Feynman diagrams are illustrated in Figure 3 for a three-level ladder system for the rephasing (top row) and non-rephasing (bottom row) pulse sequences in Figure 2. Diagrams 1, 2, and 3 corresponding to ground-state bleaching (GSB), stimulated emission (ESE), and excited-state absorption (ESA) nonlinearities, respectively.

### 2.3. 2DCS in the rephasing and non-rephasing pulse sequences

The arrival time of the conjugated pulse determines the quantum pathways accessible in the MDCS experiments. For a rephasing pulse sequence in which the conjugated pulse $\varepsilon_{1}^{*}$ arrives at the sample first, shown in Figure 2(a) and 2(b), homogeneous and inhomogeneous contributions to the optical lineshapes can be separated, as described by the following. In an inhomogeneously broadened system, each emitter *j* in the ensemble will oscillate at its resonance frequency *ω_j_* after the interaction with the first pulse. The inhomogeneous distribution of resonance frequencies *σ* leads to destructive interference of the first-order coherence of each emitter and free induction decay of the macroscopic polarization within a time $T_{2}^{*}=\hbar /\sigma$; however, after a time τ_1_, the interaction with pulses *ε*_2_ and *ε*_3_ generates third-order coherences that oscillate with opposite phase during delay τ_3_, resulting in eventual constructive interference and rephasing of the individual oscillators and the emission of a ‘photon echo’ at a time τ_3_ = τ_1_. The temporal width of the echo is inversely proportional to *σ*, while the amplitude of the echo decays at a rate equal to the ensemble-average homogeneous width *γ*. Thus, measurements of the rephasing FWM signal versus delays τ_1_ and τ_3_ provide details of both the inhomogeneous and homogeneous broadening in the system.

**Figure 4:**
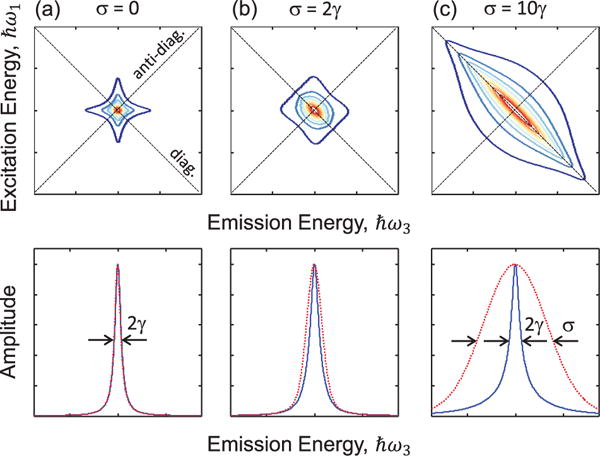
Simulated rephasing one-quantum spectrum for ratios of inhomogeneous (*σ*) to homogeneous (*γ*) broadening of (a) zero, (b) 2, and (c) 10. Lineshapes along the diagonal (ℏ*ω*_1_ = − ℏ*ω*_3_) and anti-diagonal directions are shown in the bottom row by the dashed and solid lines, respectively.

Extracting both *σ* and *γ* from a time-domain FWM experiment is often impossible without a priori knowledge of the amount of inhomogeneous broadening [46]. This ambiguity is circumvented in a 2D rephasing spectrum, shown in Figure 4 for three different ratios of *σ*/*γ* for density matrix simulations of a simple two-level system. The excitation and emission energy axes correspond to the first- and third-order coherences with energies ℏ*ω*_1_ and ℏ*ω*_3_ (*ω*_1_is considered negative for the rephasing time-ordering and thus the excitation energy increases towards the bottom of the vertical axis). A peak lineshape analysis using the projection-slice and projection-shift theorems of Fourier transforms provides analytical expressions of the diagonal (*S_D_*) and anti-diagonal (*S_AD_*) and profiles

(5) $$S_{D}=\frac{\sqrt{2\pi }}{\gamma }\mathrm{Voigt}(\gamma ,\sigma )$$

(6) $$S_{AD}=\frac{e^{-{\left(\gamma -i\omega \right)}^{2}/2\sigma^{2}}\mathrm{Erfc}\left(\frac{\gamma -i\omega }{\sqrt{2\sigma }}\right)}{\sigma (\gamma -i\omega )},$$

where the *Voigt* profile is a convolution of Gaussian and Lorentzian functions and *Erfc* is the complementary error function. These expressions allow for quantitative analysis of the inhomogeneous and homogeneous linewidths simultaneously [47,48]. They take on the form of Gaussian and square-root Lorentzian lineshapes, respectively, in the limit that *σ* ≫ *γ*.

**Figure 5:**
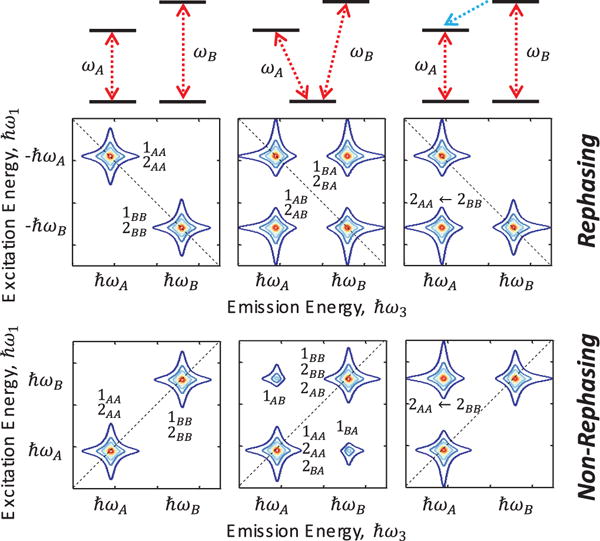
Simulated rephasing and non-rephasing one-quantum spectra for (left) two independent two-level systems, (middle) a three-level *V*-system, and (right) two independent two-level systems with incoherent energy transfer. The quantum pathway from Figure 3 contributing to each peak is labeled in the spectrum.

In the non-rephasing pulse sequence, in which the conjugate pulse arrives second, the macroscopic polarization decays at the sum of the inhomogeneous and homogeneous dephasing rates. A non-rephasing 2D spectrum can provide ancillary information that helps separate and quantify overlapping quantum pathways contributing to the nonlinear response. As an illustrative example, three different energy level systems and corresponding simulated rephasing and non-rephasing one-quantum spectra are shown in Figure 5. For two-independent two-level systems, the spectra feature two peaks on the diagonal dashed line at energies ℏ*ω*_A_ and ℏ*ω*_B_ that correspond to GSB and ESE nonlinearities for each transition. In the case of a three-level *V*-system, off-diagonal cross peaks provide clear signatures of coherent coupling between the transitions, which is expected since they share a common ground state. These peaks arise from GSB and Raman-type ESE pathways, which are low frequency, non-radiative coherences between the transitions. In the non-rephasing spectrum, the Raman-like coherence pathways overlap with the diagonal GSB and ESE peaks. In the case of two-independent transitions coupled by incoherent population transfer from the higher-to-lower state, only a single off-diagonal peak appears at the excitation energy of ℏ*ω*_B_ and emission energy of ℏ*ω*_A_.

In both rephasing and non-rephasing pulse sequences, the first two fields generate second-order populations and non-radiative Raman-type coherences, the dynamics of which are accessible during the delay τ_2_. Fourier transformation with respect to τ_2_ generates a zero-quantum spectrum, which can further separate the quantum pathways associated with these processes. While zero-quantum spectra will not be discussed in great detail in this review, the information gleaned from these types of experiments have provided unique insight into long-lived population dynamics and non-radiative coherences in semiconductor QWs [49], QDs [50], and monolayer transition metal dichalcogenides [51].

For a pulse time-ordering in which the conjugated field $\varepsilon_{3}^{*}$ arrives at the sample last, which corresponds to ‘negative’ delays in two-pulse FWM experiments, the signal should be zero according to the optical Bloch equations for an ensemble of two-level systems [52]. Experimentally, this often is not the case for semiconductors, and an anomalous negative delay signal has been a ‘smoking gun’ of coherent many-body interactions. For this pulse sequence, the first two fields generate a non-radiative two-quantum coherence between the ground and doubly excited states – either real electronic transitions or those arising from many-body interactions. The interaction of the third pulse drives the system back to a radiative one-quantum coherence that is detected as the FWM signal. Initial observations of negative-delay signals were attributed to local field effects [53,54] in which the re-radiated field from the polarization induced in the medium by the first two pulses *ε*_1_ and *ε*_2_ can drive the system after the pulses are gone. The free induction decay can persist long after the conjugated pulse $\varepsilon_{3}^{*}$ arrives, thereby producing a signal. The presence of strong excitation effects, including excitation induced dephasing (EID) and excitation induced shift (EIS), also constitute many-body phenomena that can give rise to negative-delay signals. EID [55,56] and EIS [57] are responsible for scattering of the first-order optical coherence by the second-order population induced by the third pulse into the direction of the FWM signal.

Many-body effects are challenging to treat theoretically, since the FWM signals due to EID and EIS are intrinsically non-perturbative [58]. Two-quantum FWM experiments have been particularly useful for investigating Coulomb correlations beyond the Hartree-Fock limit in which interactions are not treated perturbatively [59]. Higher order correlations responsible for bound and unbound biexcitons and signatures of exciton interactions at extremely low densities have been observed in two-quantum spectra of semiconductor QWs [60–62]. Two-quantum spectroscopy of excitons in DQWs and exciton-polaritons in microcavity QWs is discussed in more detail in Sections 4 and 6, respectively.

### 2.4. Experimental implementation of MDCS

The advantages of MDCS stem from its ability to explicitly track the amplitude *and phase* of the nonlinear optical response across multiple time periods. Phase-sensitive measurements of the FWM signal *S_i_*(*τ*_1_, *τ*_2_, *τ*_3_), where *i* denotes the order of the conjugate pulse, are possible through heterodyne interferometry between the signal and a local-oscillator reference pulse. Numerous methods exist for converting time-domain FWM signals into a multi-dimensional coherent spectrum, all of which require subwavelength phase stability and interferometric precision of the pulse delays to reliably perform numerical Fourier-transform algorithms. These schemes include passive stabilization with common optical paths [63–68] and active stabilization with feedback loops to suppress mechanical fluctuations that introduce phase noise [69–71]. Most methods are based on these two approaches with additional capabilities including single-shot measurements [72,73], broadband continuum operation [74,75], photocurrent generation and detection [23,76,77], and sub-diffraction imaging with photoemission nanoscopy [78]. In any implementation, the FWM signal must be separated from other contributions to the polarization. In this section, we present two methods for isolating the signal using non-collinear and collinear pulse-propagation geometries.

#### 2.4.1. Non-collinear geometries

**Figure 6:**
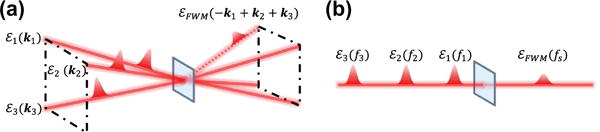
Pulse propagation geometries for MDCS experiments. (a) Non-collinear ‘box’ geometry in which three pulses *ε*_1_, *ε*_2_ and *ε*_3_ are focused onto the sample with wavevectors ***k***_1_, ***k***_2_, and ***k***_3_, respectively. The four-wave mixing signal field *ε_FWM_* is measured in the phase-matched direction ***k****_S_* = −***k***_1_ + ***k***_2_ + ***k***_3_. (b) Collinear geometry in which the four-wave mixing signal can be isolated through dynamic phase-cycling. Zero-, one-, and two-quantum spectra are measured by varying the time-ordering of the conjugate pulse in (a) or detecting the signal at a different radiofrequency in (b).

The most routinely employed configuration for MDCS techniques is background-free transient FWM, which is shown for the ‘box’ geometry in Figure 6(a). In this configuration, three pulses *ε*_1_, *ε*_2_, and *ε*_3_ propagating parallel to each other along the three corners of a virtual box are focused onto the nonlinear medium with wavevectors ***k***_1_, ***k***_2_, and ***k***_3_, respectively. Due to conservation of momentum, the transient FWM signal radiates in the phase-matched directions ***k***_S1_ = −***k***_1_ + ***k***_2_ + ***k***_3_, ***k***_S2_ = ***k***_1_ − ***k***_2_ + ***k***_3_, and ***k***_S3_ = ***k***_1_ + ***k***_2_ − ***k***_3_ for the rephasing (*S*_1_), non-rephasing (*S*_2_), and two-quantum (*S*_3_) signals, respectively, as indicated in the pulse diagrams in Figure 2. In principle, each component can be measured simultaneously by detecting the signal in the corresponding phase-matched direction. In practice, the same result is achieved by monitoring the signal in the direction ***k***_S1_ only and then performing multiple measurements with different time-ordering of the conjugate pulse to acquire each component.

The FWM signal amplitude and phase are acquired through heterodyne interferometry with a fourth reference pulse with a known spectral amplitude and phase [79,80]. The reference pulse is usually routed around the nonlinear medium to avoid photo-exciting additional carriers in system. The reference-signal interference is spectrally resolved to obtain the emission energies of the signal, *S_i_*(*τ*_1_, *τ*_2_, ℏ*ω*_3_). Scanning τ_1_ and taking a Fourier transform with respect to this delay generates a one-quantum rephasing/non-rephasing spectrum *S*_1/2_(ℏ*ω*_1_, *τ*_2_, ℏ*ω*_3_). Alternatively, scanning the delay τ_2_ generates a zero-quantum spectrum *S*_1_(*τ*_1_, ℏ*ω*_2_, ℏ*ω*_3_), or two-quantum spectrum *S*_3_(*τ*_1_, ℏ*ω*_2_, ℏ*ω*_3_) for the rephasing and two-quantum pulse sequences, respectively.

Several platforms have been developed to produce four identical pulses in the ‘box’ geometry. One comprises a series of nested Michelson interferometers with translation-stage delay lines and either passive phase-stabilization with common-path optics [68] or active stabilization with a continuous-wave reference laser and servo-loops feeding back onto piezo-electric actuators to compensate the mechanical phase noise [70,71]. These approaches offer the most versatility in extracting all pulse sequences with pulse delays limited only by the maximum travel of the translation stages, but at the expense of experimental complexity. Temporal resolution *δτ* of ~1 fs and maximum scan range Δ*τ* greater than 1 ns are readily achievable. A clever alternative for generating a non-collinear pulse sequence is through diffractive optics and spectral pulse shapers, which ensures optimal passive phase stabilization [63,66,67]. The diffracted beams and pulse delays are provided by separate spatial light modulators, which also enable independent control the spectrum of each pulse [13]. Compared to interferometric approaches, pulse-shaping reduces the complexity of experiments, since moving parts are eliminated; however, the maximum achievable delay is Δ*τ* ≈ 10 ps, which corresponds to a spectral resolution of ~500 μeV (100 GHz, ~3 cm^−1^). Consequently, such approaches are unable to probe the slow dynamics of many semiconductor heterostructures, such as QWs (~tens of picoseconds) and QDs (~nanoseconds).

These techniques can be extended into three dimensions by scanning both τ_1_ and τ_2_ to generate a three-dimensional spectrum *S_i_*(ℏ*ω*_1_, ℏ*ω*_2_, ℏ*ω*_3_), which provides maximal separation of the quantum pathways up to χ^(3)^ [36]. A general problem encountered in 3D experiments is the long data acquisition time, which scales as *N*_1_*N*_2_, where *N_i_* = **Δ***τ_i_*∕*δτ* is the number of points acquired for delay. This situation is mitigated by exploiting under-sampling techniques in which the signal is acquired at a sampling rate *f_S_* = 1/*δτ* that can be well-below the Nyquist frequency *f_N_* = 2*f*, where *f* is the largest frequency component in the signal. An alias of the under-sampled signal will appear at frequencies *f_A_* = |*f* – 2*nf_S_*|, where *n* is an integer. Full reconstruction of the FWM signal is possible by carefully choosing *f_S_* so that no spurious or unrelated signals are present within the acquisition bandwidth centered at *f_A_*. Compressed-sensing techniques have also been effective at enhancing the data acquisition rate through random sparse sampling algorithms [81].

#### 2.4.2. Collinear geometries

While non-collinear geometries can isolate FWM signals from extended media on the order of the optical wavelength λ or larger through phase-matching, conservation of momentum does not apply for nanoscale objects because of the lack of translational symmetry. A single emitter Δ*x* in spatial extent will radiate into a wavevector distribution Δ*k* ≈ 1∕Δ*x*; for typical nano-emitters such as QDs, the emission is essentially isotropic. Collinear approaches circumvent this problem through frequency-modulation and phase-cycling techniques that are the frequency-domain analog of wavevector phase matching [82].

In collinear MDCS experiments (see Figure 6(b)), the frequency of the *i*th pulse is shifted by a unique radio frequency *f_i_* using an acousto-optic modulator (AOM). This scheme is akin to dynamic phase cycling in which the carrier-envelope phase of pulse-train *ε_i_* is cycled at *f_i_*. Phase modulation of the beams is imparted onto the frequency *f_S_* of the FWM signal as shown in Figure 2, resulting in signal frequencies *f_S_*_1_ = −*f*_1_ + *f*_2_ + *f*_3_ for the rephasing one- and zero-quantum sequence, *f_S_*_2_ = *f*_1_ − *f*_2_ + *f*_3_ for the non-rephasing sequence, and *f_S_*_3_ = *f*_1_ + *f*_2_ − *f*_3_ for the two-quantum sequence. The FWM signal can be detected by mixing it with a reference beam using an additional AOM, which diffracts them both into the same direction for heterodyne spectral interferometry [83]. An advantage of the collinear configuration is the ability to use a high-numerical aperture microscope objective to achieve diffraction-limited focusing and high-efficiency collection of the signal. High spatial resolution facilitates hyperspectral mapping of the FWM signal, which has been implemented to study individual QDs in an ensemble [11,84] (see Section 4). The FWM signal can also be detected using the reference beam to generate a fourth-order population that is collected in fluorescence with a single-element detector. The main advantage of this approach is that the detector signal contains all the radio-frequency beat notes for each pulse sequence, which can be independently demodulated and measured in a single experiment [85,86]. The concepts from fluorescence spectroscopy have been recently adapted to detect photocurrents in semiconductor diode nanostructures [23,76,77], discussed in Section 7. For these types of collinear experiments, undersampling is important for achieving realistic data acquisition rates because the emission energies ℏ*ω*_3_ are now obtained by scanning the delay τ_3_ between the reference and *ε*_3_ followed by a numerical Fourier transform instead of a spectrometer.

## 3. Semiconductor quantum dots

**Figure 7:**
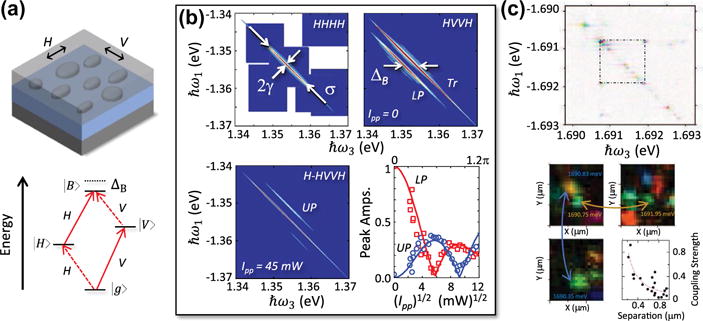
2DCS of semiconductor quantum dots. (a) Cartoon of a semiconductor quantum dot ensemble (top). The lowest energy exciton (|H⟩ and |V⟩) and biexciton (|B⟩) transitions are represented by a four-level diamond energy diagram. (b) Rephasing one-quantum spectra of a self-assembled InAs/GaAs ensemble measured at 5 K for co-linearly (*HHHH*) and cross-linearly (*HVVH)* polarized optical excitation and detection. The homogeneous and inhomogeneous lineshapes are separated along the anti-diagonal and diagonal directions, yielding an exciton homogeneous width *γ* ≈ 10 μeV for *HHHH*. For *HVVH*, the diagonal peak (*Tr*) corresponds to the trion transition in charged quantum dots and the off-diagonal peak (*LP*) is associated with bound biexcitons. Following excitation with a horizontally polarized pre-pulse with 45 mW average power arriving 15 ps before the 2D excitation pulse sequence, signatures of Rabi flopping appear as an upper off-diagonal peak (*UP*). The off-diagonal peak amplitudes (symbols) and simulated Rabi oscillations (lines) versus pre-pulse power are shown in the bottom-right panel. (c) Top: Rephasing one-quantum spectrum of a few dots in an interfacial GaAs/AlGaAs quantum dot ensemble. Off-diagonal peaks reveal coherent coupling between two dots. Bottom: Hyperspectral images of the four-wave mixing signal revealing the inter-exciton separation between coupled quantum dots.

Semiconductor QDs are an exemplary system to demonstrate the capabilities of 2DCS for dissecting disordered ensembles. The stochastic nature of the epitaxial growth process leads to an inhomogeneous distribution of QD sizes that translates into ~50–100 meV dispersion of the exciton transition energies for self-assembled InAs/GaAs QDs and ~1–5 meV for ‘natural’ GaAs/AlGaAs QDs. The in-plane QD confinement potential tends to be asymmetric with principle axes along the [110] ≡ *V* and $[1\bar{1}0]\equiv H$ crystal directions due to several sources, including strain, shape, and piezoelectricity [50,87–89] (see Figure 7(a)). When considering the electron-hole exchange interaction, anisotropy mixes the electronic transitions, resulting in two bright exciton states |*H*〉 and |*V*〉 that are separated in energy by the so-called fine-structure splitting.

To measure the exciton homogeneous linewidth (*γ*) and fine-structure splitting, optical studies originally required isolating single QDs due to inhomogeneous broadening. Using photoluminescence spectroscopy, the exciton linewidth and emission polarization properties have been characterized, and multi-particle states associated with charged excitons and biexcitons have been identified [90–92]. In single-dot studies, characterizing how statistical fluctuations within the ensemble affect the optical properties requires repeating multiple measurements on different QDs, and details of inter-dot interactions are not accessible. Alternatively, ensemble measurements are possible with nonlinear spectroscopy techniques [93,94]. Transient FWM studies have been useful for characterizing the exciton dephasing time (*T*_2_ = ℏ/*γ*) and radiative lifetime (*T*_1_). Oscillations of the FWM signal have also been identified as coherent quantum beats between |*H*〉 and |*V*〉 and |*H*〉 and |*B*〉. Despite the rich dynamics identified by these studies, transient FWM is not sensitive to dot-to-dot fluctuations of the homogeneous properties because it provides an ensemble-averaged response.

Optical 2DCS offers the specificity of single-dot studies while probing the entire ensemble. This capability is illustrated by the rephasing spectrum shown in Figure 7(b) for an InAs QD ensemble at 10 K, where the emission energies are plotted as negative for this pulse sequence [95,96]. The spectrum is shown for collinearly polarized excitation and detection aligned along *H* (denoted as an *HHHH* polarization scheme corresponding to the polarization of pulses *ε*_1_, *ε*_2_, *ε*_3_ and the signal). The single peak on the diagonal dashed line is attributed to GSB and ESE of the |*H*〉 exciton transition. The large linewidth along the diagonal compared to the anti-diagonal direction indicates that material is strongly inhomogeneously broadened (*σ* ≫ *γ*). Anti-diagonal slices as a function of exciton resonance energy throughout the inhomogeneous distribution reveals a relatively uniform homogeneous linewidth *γ* ≈ 10 μeV.

For an *HVVH* polarization scheme, the GSB and ESE quantum pathways for the exciton state are no longer accessible. In this case, the rephasing spectrum features two peaks: the diagonal peak *Tr* is associated with positively charged excitons in charged QDs within the ensemble, and the off-diagonal peak *LP* corresponds to ESA to the biexciton state |*B*〉 in neutral QDs. The shift of *LP* to lower energy along ℏ*ω*_3_ provides a measure the biexciton binding energy Δ*_B_* relative to *E_H_* + *E_V_*. Interestingly, the binding energy seems to be similar for the entire ensemble, which is attributed to thermal annealing of the sample that homogenizes the QD properties.

The ability of 2DCS to resolve different frequency groups within the ensemble also allows the technique to serve as a sensitive probe of quantum states in coherent control experiments of ensembles [10]. For the *HVVH* polarization scheme, pre-excitation of the ensemble with a strong excitation pulse polarized along *H* initializes populations into the |*H*〉 exciton state and the |*B*〉 biexciton state. Additional quantum pathways starting from these states contribute to the 2D rephasing spectrum as peak *UP* as well as at the energy of *LP* with opposite sign compared to the pathways for *LP* without the pre-pulse. Thus, *LP* represents the difference between the ground state population *ρ_g_* and the population *ρ_H_* in state |*H*〉; similarly, *UP* represents the difference between *ρ_H_* and *ρ_B_*. The populations are shown in Figure 7(b) as a function of the square root of the pre-pulse power $\sqrt{I_{pp}}$, which is proportional to the pulse area. The populations clearly display Rabi oscillations (experiment and theory indicated by the points and curves, respectively), which usually require isolation of single QDs to avoid the detrimental effects of inhomogeneity [97–101].

When just a few QDs are probed within the ensemble, evidence of coherent coupling between excitons at different energies is observed. Figure 7(c) is a rephasing spectrum from a GaAs/AlGaAs ‘natural’ QD nanostructure, where only a few QDs are probed in this case using a sparse QD sample and collinear 2DCS geometry with 600 nm spatial resolution. Coupling between excitons at energy ~1.691 eV and ~1.692 eV is evident by the off-diagonal peaks between them. An analysis of the spectral phase reveals that the nature of their coupling arises from biexcitonic interactions between two excitons. Hyperspectral spatial images of the FWM signal elucidate the spatial extent across which excitons are coupled. By correlating the FWM signal energies of coupled resonances with their spatial position, the authors find that long-range coupling of extended exciton states is also important.

## 4. Layered semiconductors

Semiconducting compounds in which the covalent bonds within the crystal plane are significantly stronger than the weak bonds between planes comprise the family of layered semiconductors. Examples including InSe, GaSe, Bi_2_Se_3_, and van der Waals materials. Excitons in single monolayers exhibit interesting physics in two dimensions, which can give rise to a host of new electronic and optical phenomena. For this review, we consider narrow GaAs QWs within this group, since they represent a canonical example of exciton physics in a quasi-2D system.

### 4.1. Double quantum wells

Although a typical 10 nm-thick GaAs QW is wider than the exciton Bohr radius (~6–7 nm), the electronic transitions are well-described by quantum mechanics in two dimensions. QW ‘layers’ separated by a narrow barrier represent an interesting model system for studying long-range interactions between layers in a well-controlled environment. The thickness and separation of each QW can be tailored to mimic processes in other systems, such as energy transfer in light-harvesting complexes.

**Figure 8:**
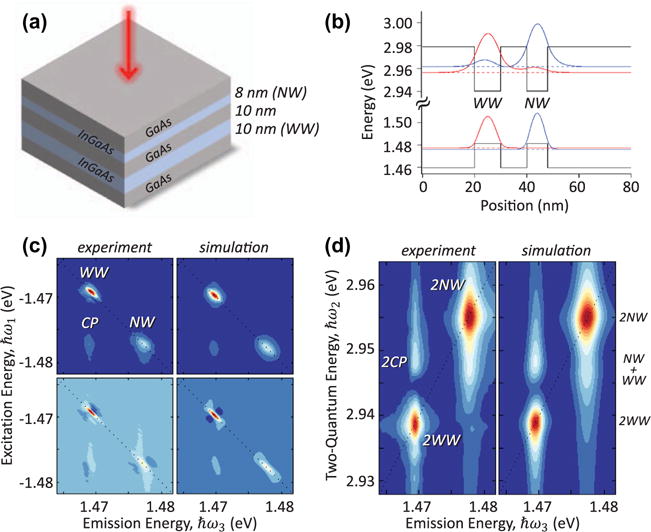
Normalized 2D spectra of an asymmetric double InGaAs/GaAs quantum well at 15 K. (a) Cartoon of the double quantum well structure. (b) Single-particle energy levels and wavefunctions calculated from the one-dimensional Schrödinger equation. (c) Magnitude (top) and real part (bottom) of the experimental (left) and simulated (right) rephasing one-quantum spectrum. The spectra feature two peaks on the diagonal associated with excitons in the narrow well (NW) and wide well (WW) and an off-diagonal coupling peak (CP). (d) Experimental (left) and simulated (right) two-quantum spectrum indicating coherent interactions between excitons confined to the two wells. Simulations are based on the optical Bloch equations with many-body interactions included phenomenologically as described in the main text.

As one of the simplest examples, semiconductor DQWs have attracted significant experimental and theoretical interest for several decades. DQWs can be realized in epitaxially grown systems, where, for example, a 10 nm-wide (*WW*) and an 8 nm-wide (*NW*) QW are separated by a barrier, as depicted in Figure 8(a). One-dimensional Schrödinger equation calculations of the single-particle electron and hole wavefunctions show that for a narrow barrier (≲ 10 nm), the hole wavefunctions are localized to their respective QWs, whereas the electron wavefunctions are partially delocalized across the two wells (Figure 8(b)). This type of coupled DQW system has been studied to understand the roles of phonon-assisted tunneling [102], carrier percolation through the barrier [103], dipole-dipole interactions [104], and resonant energy transfer in the inter-well coupling dynamics [105].

The role played by many-body effects, which govern exciton physics in a single QW, has been more difficult to identify in DQWs until recently. Using optical 2DCS, researchers have shown that inter-well coupling in an InGaAs/GaAs asymmetric DQW nanostructure originates from many-body interactions [12,106]. An analysis of zero-, one-, and two-quantum 2D spectra provides a comprehensive picture of the exciton dynamics and coupling mechanisms. In the one-quantum rephasing spectrum shown in Figure 8(c), coupling between excitons primarily localized to their respective QW is apparent from the off-diagonal peak *CP*. The real part of the spectrum (bottom panel) reveals the complex lineshape of the peak, which is particularly sensitive to many-body interactions. The dispersive lineshape is indicative that EIS plays a key role in the inter-well coupling.

Further evidence of many-body effects as the coupling mechanism is provided by the non-zero signal in the two-quantum spectrum shown in Figure 8(d). The two peaks on the diagonal line ℏ*ω*_2_ = 2ℏ*ω*_3_ arise from coherent interactions between two excitons in the same QW. The appearance of the off-diagonal peak *2CP* at the two-quantum energy *E_WW_* + *E_NW_* necessarily requires coherent coupling between excitons in the two QWs. Through density matrix calculations, it is shown that hybridization of the electronic states due to wavefunction tunneling cannot account for the interaction; instead, many-body effects are essential for understanding the coherent inter-well coupling. An analysis of the peak shapes at lower excitation densities (< 10^8^ excitons/cm^2^) than the experiments discussed above (> 10^9^ excitons/cm^2^) reveal that carrier interactions are important at lower densities than previously thought [62]. Concepts developed to understand inter-well coupling have also been helpful in characterizing interactions in other material systems including modulation-doped QWs [107].

### 4.2. Transition metal dichalcogenides

**Figure 9:**
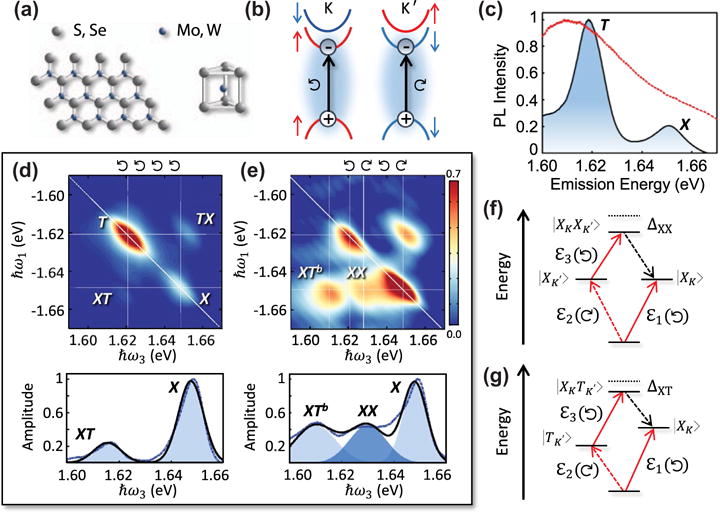
2D rephasing one-quantum spectroscopy of doped (*n*-type) monolayer transition metal dichalcogenide MoSe_2_ at 10 K. (a) Monolayer MoSe_2_ has a honeycomb crystal lattice structure with the transition metal sandwiched between two layers of chalogen atoms. (b) Band-edge direct optical transitions occur at the + *K* and −*K* (*K*′) points, or valleys, in momentum space. Transition dipole selection rules dictate that electron-hole pairs are photo-excited at the *K* and *K*′ valleys with left- (right-) circularly polarized optical excitation, respectively. (c) Photoluminescence spectrum featuring two peaks near 1.65 and 1.62 eV associated with the neutral exciton (*X*) and charged exciton (trion, *T*). The excitation spectrum for the 2D spectroscopy experiments is indicated by the dashed curve. (d) Left: The rephasing one-quantum spectrum for co-circularly polarized excitation and detection features exciton and trion peaks on the diagonal and their off-diagonal coupling peaks *XT* and *TX*. A horizontal slice near the exciton excitation energy ℏ*ω*_1_ = −1.65 eV is shown in the bottom panel with Gaussian fits. (e) Similar to (d) but for alternating circular polarization of the excitation pulses and detection. Two additional peaks *XX* and *XT^b^* are attributed to the neutral and charge biexcitons forming between two excitons or an exciton and trion in opposite valleys, respectively. The quantum pathways corresponding to *XX* and *XT^b^* are shown in (f) and (g), respectively.

Transition metal dichalcogenides (TMDs) exhibit unique electronic and optical properties at the ultimate two-dimensional limit [108–110]. Group-VI TMDs have a hexagonal crystal lattice structure with chemical formula *MX_2_* {*M* = Mo, W; *X* = S, Se, Te}, where the *M* and *X* atoms reside at the *A* and *B* lattice sites (see Figure 9(a)). Like graphene and other 2D materials, a single atomically thin monolayer can be isolated through mechanical exfoliation or grown with vapor deposition techniques. Monolayer TMDs are a direct gap semiconductor, where the minimum of the conduction band and maximum of the valence band reside at the two degenerate + *K* and −*K* (*K*′) points (dubbed ‘valleys’) in the first Brillouin zone (Figure 9(b)). The *K* and *K*′ valleys are related to each other through time-reversal symmetry; combined with large spin-orbit coupling and broken spatial inversion symmetry in monolayers, the symmetry leads to opposite electronic spin, orbital magnetic moment, and Berry curvature at the two valleys [111]. Consequently, the dipole-allowed optical transitions are also valley dependent: left- (right-) circularly polarized light excites an electron-hole pair in the *K* (*K*′) valley.

One consequence of the atomic thickness of monolayer TMDs is a significant enhancement of the Coulomb force due to reduced dielectric screening in two dimensions. Combined with the large carrier effective masses, reduced screening leads to pronounced excitonic effects with neutral and charged exciton binding energies on the order of 500 and 30 meV, respectively – an order of magnitude larger than conventional semiconductors like GaAs-based nanostructures [112]. At room temperature, excitons govern the optical response of TMDs and absorb up to 20% of the incident light. A typical photoluminescence spectrum from MoSe_2_ on a sapphire substrate at 10 K is shown in Figure 9(c). The two peaks correspond to the neutral exciton (*X*) and charged exciton (*T*) transitions (common to monolayer TMDs is unintentional doping from the substrate that is responsible for the trion peak).

Although monolayer TMDs are relatively new to the large family of layered semiconductors, a large body of work already exists examining the origin, dynamics, transport, and interactions of excitons [113–117]. Recent 2DCS experiments have added to this knowledge-base through measurements of the coherent exciton dynamics and many-body interactions. A rephasing one-quantum spectrum of monolayer MoSe_2_ at 10 K on a sapphire substrate is shown in Figure 9(d) for co-circular excitation and detection [18]. Several important details are immediately evident from this spectrum. First, the exciton (*X*) and trion (*T*) resonances exhibit moderate inhomogeneous broadening due to disorder potentials possibly from impurities, defects, and strain in the system. At low temperature and density, the homogeneous linewidths are on the order of 1 meV. A comparison to the population lifetime reveals that the exciton coherence time is lifetime limited (*T*_2_ = 2*T*_1_), whereas trion decoherence is significantly faster than its lifetime (*T*_2_ ≪ *T*_1_). This observation might be attributed to a greater susceptibility for trion interactions with the Fermi sea or charged surface adsorbates and defects in the monolayer. Second, an analysis of the off-diagonal peaks reveals coherent coupling and quantum beats between *X* and *T* that arise from EIS between excitons and trions [17,118].

The rephasing spectrum is quite different for cross-circularly polarized excitation and detection in which excitons and trions in both valleys are now accessible (Figure 9(e)). The key feature is the observation of a new off-diagonal peak (*XX*) that has been assigned to the bound biexciton with a binding energy Δ*_XX_* = 2ℏ*_X_* − 2ℏ*_XX_* = 20 meV along the emission energy axis ℏ*ω*_3_. Additionally, the below-diagonal peak (*XT^b^*) shifts to lower emission energy by $\Delta_{XT}=\hbar \omega_{X}+\hbar \omega_{T}-\hbar \omega_{XT^{b}}=5\;\text{meV}$ relative to the co-circularly polarized spectrum, which is consistent with a bound charged biexciton state. Both binding energies extracted from the spectra are in reasonable agreement with current theoretical models [119,120]. Furthermore, these states only appear for cross-circular polarization, indicating that they originate from intervalley coupling between excitons and trions. The quantum pathways for these two peaks are shown in Figure 9(f) and 9(g), respectively. It is worth noting that the spectral proximity of *XX* and *XT^b^* emission with *T* makes them difficult to access in one-dimensional spectroscopy techniques, whereas they are clearly separated in the 2D spectra as shown by the horizontal slices taken along ℏ*ω*_3_ at the exciton excitation energy ℏ*ω*_1_ = −1.648 eV.

2DCS experiments of other TMD materials (WSe_2_ and MoS_2_) have revealed rich exciton dynamics arising from non-radiative coherences between opposite valley states [51], exciton-phonon and exciton-exciton interactions [20,21], and coupling between the exciton and electron-hole continuum [19]. 2DCS has also helped identify the mechanism of exciton dephasing in layered InSe [121] and energy transfer between singlet and triplet states in layered GaSe [122].

### 4.3. Semiconducting carbon nanotubes

**Figure 10:**
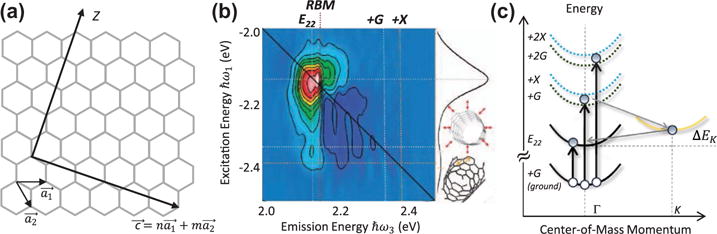
(a) Hexagonal lattice structure of a nanotube with real-space unit vectors $\vec{a_{1}}$ and $\vec{a_{2}}$. The vector $\vec{c}$ denotes the chirality of the nanotube, where *Z* is the tube axis. (b) Real part of a 2D rephasing spectrum from a (6,5) single-walled carbon nanotube under ambient conditions for τ_2_ = 100 fs. Population transfer between the diagonal exciton (*E*_22_) and phonon sideband states (*G* and *X*) is revealed by the off-diagonal cross peaks. (b) Energy level diagram depicting absorption quantum pathways of pulse *ε*_3_ from the ground-state vibrational coherence of the *G*-band mode induced by stimulated Raman-type processes from the first two pulses. Excitation from the *G*-band (ground) to the excited-state phonon sidebands is accompanied by phonon-mediated relaxation to the *E*_22_ exciton state.

Single-walled carbon nanotubes (SWNTs) possess many unique electrical and optical properties that make them appealing for next-generation opto-electronics and photonics [123]. The photo-physics of quasi one-dimensional excitons confined along the circumferential direction are highly tunable via the configuration and diameter of the SWNT [124]. The structure can be conceptualized by wrapping a monolayer sheet of graphene into cylindrical form along an axis *Z* defined by a pair of chiral indices (*n*,*m*) (see Figure 10(a)) that correspond to the number of unit vectors $\vec{a_{1}}$ and $\vec{a_{2}}$ in the real-space honeycomb crystal lattice. The properties of a SWNT are highly dependent on its chirality. In general, for |*m* − *n*| = 3*k*, where *k* is an integer, the SWNT is metallic; otherwise, the SWNT is a semiconductor, although there are exceptions to this rule [125]. The optical spectrum of a single semiconductor SWNT contains a series of sharp peaks (denoted *E*_11_, *E*_22_, etc.) whose energy scales inversely with the tube diameter $d=\left(|\vec{a_{1}}|/\pi \right)\sqrt{n^{2}+nm+m^{2}}$, which is on the order of ~1 nm. The ability to control the optical bandgap and electrical behavior makes SWNTs a model system for studying one-dimensional exciton and charge transfer physics.

Films containing a mesoscale network of interwoven SWNTs are readily synthesized with solution-based processing techniques [126]. A purification processing step enables sorting of semiconducting nanotubes from metallic ones that detrimentally quench exciton emission. The energetic manifold of a SWNT film, which comprises an ensemble with many different chiralities, is extremely complex, since there are multiple discrete transitions for each structure type. Steady-state photoluminescence excitation (PLE) spectroscopy has been the predominant method for resolving the different chiralities and energy transfer between them; however, without any time-resolution, PLE cannot distinguish between different energy transfer pathways that lead to emission at the same energy, even for an isolated SWNT [127].

Several variants of broadband optical 2DCS have been developed to study these dynamics. The results from one such experiment is shown in the one-quantum rephasing spectrum in Figure 10(b), which corresponds to excitation and emission near the *E*_22_ transition of an aqueous suspension of (*6,5*)-enriched SWNTs [22]. The strongest diagonal feature corresponds to the ESE and GSB pathways of the *E*_22_ exciton transition. The off-diagonal peaks explicitly show that excitons also couple to several prominent phonon modes, including the radial breathing mode (RBM at 296 cm^−1^, ~37 meV) and the *G*-band (1590 cm^−1^, ~197 meV). An analysis of the dynamics of the off-diagonal peaks during delay *τ*_2_ further elucidates their origin. Oscillations of the peak amplitudes with periods of ~21 fs and ~110 fs indicate that impulsive optical excitation creates a coherent wavepacket corresponding to a vibrational superposition of the exciton with the *G*-band (21 fs) and the RBM (110 fs) through stimulated Raman-type processes. The off-diagonal peaks are also enhanced by multi-phonon scattering involving higher energy *X*-band modes and the *K*-point state. This pathway is depicted in Figure 10(c).

2DCS experiments have also found utility for understanding energy transfer in mixed ensembles with multiple chiralities. Energy transfer from higher bandgap to lower bandgap SWNTs ranging from 10’s of femtoseconds between bare nanotubes to several picoseconds for nanotubes wrapped in a conjugated polymer coating has been identified [128,129]. Packing SWNTs with synthetic heterodimers also serves as a model system for emulating natural light-harvesting complexes; observations of quantum beating in the 2D spectra from these SWNTs have helped elucidate the origin of energy transfer in photosynthesis [130].

## 5. Three-dimensional spectroscopy of quantum wells

By extending 2DCS into three dimensions, the different quantum pathways can be further separated to reveal hidden details of the mechanisms contributing to a system’s nonlinear optical response. 3DCS techniques have been developed in the infrared to examine vibrational and structural relaxation dynamics of molecules [131–133] and extended to the visible regime to resolve high-frequency vibronic coherences [134], to fully distinguish all quantum pathways in an atomic vapor up to χ^(3)^ [36], and to separate pathways involving weakly bound mixed-biexciton states in a GaAs QW [135].

When applied to semiconductor nanostructures, 3DCS is an effective tool for resolving ultrafast electronic dynamics and weak optical transitions that are otherwise obscured in a 2D spectrum. In an asymmetric InGaAs DQW nanostructure similar to the one discussed in Section 4.1, 3DCS experiments have revealed new transitions and inter-well coupling mechanisms that are difficult to identify with other optical techniques [136–138]. Weak optical transitions associated with excitons delocalized within the QW barriers, spatially indirect excitons with an electron in one QW and the hole in the other, and parity-forbidden excitons have been observed; interactions between them lead to a complex 3D spectrum with at least 5 diagonal peaks and 18 off-diagonal peaks [139]. A quantitative analysis of the 3D spectral lineshapes provides new insight into their coupling mechanisms.

**Figure 11:**
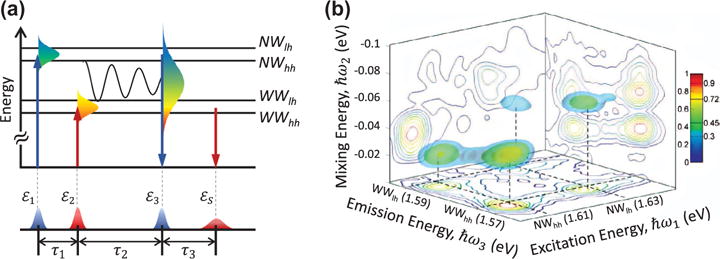
3D rephasing spectrum (amplitude) of an asymmetric double InGaAs quantum well nanostructure at 20 K for collinearly polarized excitation. (a) Shown is the optical excitation sequence with spectrally-shaped pulses to isolate specific off-diagonal peaks corresponding to Raman-type coherence quantum pathways between the *WW* and *NW*. (b) 3D spectrum revealing off-diagonal coherence peaks fully isolated in three-dimensions allowing for quantitative peak shape analysis.

A specific interaction process can be further isolated in a 3D spectrum by selectively exciting and probing specific quantum pathways through spectral shaping of the pulses [13]. Multi-color 3DCS has been demonstrated on the asymmetric InGaAs DQW system to identify, isolate, and analyze weak coherent coupling between excitons in spatially separated QWs. The two-color pulse sequence is shown in Figure 11(a). The spectrum of the first pulse *ε*_1_ is filtered so that it is resonant only with the exciton transitions in the *NW*. Similarly, the second pulse *ε*_2_ is resonant only with the *WW* transition. The interaction of these two pulses with the DQW drives the system into a non-radiative Raman-like coherence between the *WW* and *NW* states that evolves during the delay τ_2_. The third pulse *ε*_3_ has the full spectral bandwidth to excite transitions in both QWs. Using this pulse sequence, the quantum pathways arising from the inter-well stimulated Raman coherence are isolated in the 3D spectrum (Figure 11(b)). The absence of other quantum pathways enhances the signal-to-noise of these peaks, enabling quantitative peak lineshape analysis. The ability to isolate specific quantum pathways using the combination of spectral shaping and 3DCS might help to resolve open questions regarding the role of quantum mechanics in the energy transfer processes of photosynthetic complexes and other material systems, including layered semiconductors.

## 6. Exciton-polaritons in semiconductor microcavities

**Figure 12:**
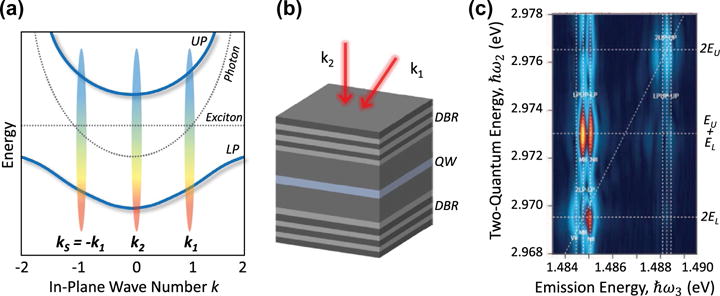
Two-quantum spectrum (magnitude) of exciton-polaritons in a single In_0.04_Ga_0.96_As quantum well in a GaAs/AlGaAs microcavity at 4 K. (a) Diagram depicting the lower polariton (*LP*) and upper polariton (*UP*) dispersion curves for a negative cavity-exciton detuning. The dashed black curves correspond to the bare exciton and cavity energy-momentum dispersion. The pump, probe, and signal wavevectors are indicated. (b) Semiconductor microcavity structure and excitation pulse geometry in the pump-probe configuration, with k_2_ = 0 μm^−1^ and k_1_ = 0.96 μm^−1^. (c) Two-quantum spectrum revealing coherent cross-interactions between upper- and lower-polaritons (peaks at ℏ*ω*_2_ = *E_U_* + *E_L_*) and coherent self-interactions between lower polaritons (at ℏ*ω*_2_ = 2*E_L_*) and upper polaritons (at ℏ*ω*_2_ = 2*E_U_*).

The resonant interaction between semiconductor excitons and an optical field inside a microcavity can strongly modify the light-matter interaction. In the strong coupling regime, the exciton and cavity modes form new admixed eigenstates known as upper (*UP*) and lower (*LP*) exciton-polaritons that exhibit an anti-crossing in their dispersion curves (see Figure 12(a)). Exciton-polariton states can be created by embedding a QD or a QW within a semiconductor microcavity [140]. A typical group *III*–*V* microcavity system consists of a single or few layers of In_x_Ga_1–x_As QWs sandwiched between GaAs/AlAs distributed Bragg reflectors (DBRs) acting as the cavity end mirrors. Due to their mixed light-matter character, exciton-polaritons have extremely low effective mass that facilitates Bose-Einstein condensation [141] and superfluidity [142] in the solid-state. Because they retain an excitonic component, exciton-polaritons are strongly influenced by the many-body interactions inherent to semiconductor nanostructures. Transient FWM experiments reveal that two-exciton Coulomb correlations clearly play a role in exciton-polariton scattering and decoherence [143–146].

Substantial progress in quantitatively understanding the details of higher order correlations and coherent exciton-polariton scattering has been attained in the last few years with non-collinear optical 2DCS techniques. A typical experimental configuration is shown in Figure 12(b), where two excitation pulses *ε*_1_ and *ε*_2_ with wavevectors ***k***_1_ and ***k***_2_ interact with the nanostructure to generate a nonlinear signal in the phase-matched direction 2***k***_2_ − ***k***_1_ (FWM), 3***k***_2_ − 2***k***_1_ (six-wave mixing), or 4***k***_2_ − 3***k***_1_ (eight-wave mixing). A representative two-quantum 2D spectrum obtained for the ‘negative’ time-ordering is shown in Figure 12(c). The experiment is performed on a microcavity containing a single 8-nm wide In_0.04_Ga_0.96_As QW with a cavity-exciton detuning δ = −0.38 meV and a sample temperature of 4 K [14]. The wave numbers of the excitation beams and the FWM signal are shown schematically in Figure 12(a). The fact that the two-quantum signal is non-zero indicates that two-polariton interactions are coherent. The grouped peaks on the diagonal near 2*E_L_* and 2*E_U_* arise from lower and upper polariton-polariton self-interactions, respectively, that lead to a slight blue-shift of the two-polariton state compared to the sum of the individual polariton energies. Similarly, the off-diagonal peaks arise from polariton-polariton cross-interactions. Non-perturbative numerical simulations of the FWM signal attribute the fine structure within each peak group to non-resonant polariton-polariton scattering within the energy-momentum dispersion curves.

Interestingly, higher order multi-quantum 2D spectra from six- and eight-wave mixing experiments reveal that up to at least four exciton-polaritons can be correlated – a higher order than what has been observed in bare QWs, demonstrating that higher order correlations are mediated via the cavity [15]. A map of the cavity-exciton detuning on the relative peak amplitudes, homogeneous dephasing rate, and inhomogeneous broadening from the 2D spectra provides additional insight into how Coulomb correlation strengths are modified as the lower and upper polariton branches intersect [16]. Applying similar methods to study coherent many-body phenomena in other material systems that exhibit strong excitonic interactions and light-matter coupling, such as transition metal dichalcogenides, might facilitate the realization of polariton condensation and superfluidity at room temperature.

## 7. Two-dimensional photocurrent spectroscopy of nanostructures

An enticing alternative approach to the conventional optical heterodyne-detection techniques of MDCS is to instead use a sequence of *four* collinear pulses to drive a fourth-order population in the system. The resulting excited-state populations can be detected either optically as fluorescence, which is the basis for 2D fluorescence spectroscopy previously demonstrated on a rubidium vapor [86], or through photocurrent in a semiconductor photoconductive device [76]. Isolation of the fourth-order population from other signals is achieved by utilizing acousto-optic phase modulation of the excitation pulses discussed in Section 2.4.2 in combination with phase-synchronous detection using a lock-in amplifier. Mechanical fluctuations in the setup can be partially compensated for using an auxiliary continuous-wave laser running through the same optics as the excitation laser pulses, which eliminates the need for active phase stabilization schemes. This approach offers several advantages compared to conventional MDCS methods, including simultaneous detection of the rephasing, non-rephasing, and two-quantum signals. Due to the collinear geometry, diffraction-limited spatial resolution is possible.

**Figure 13:**
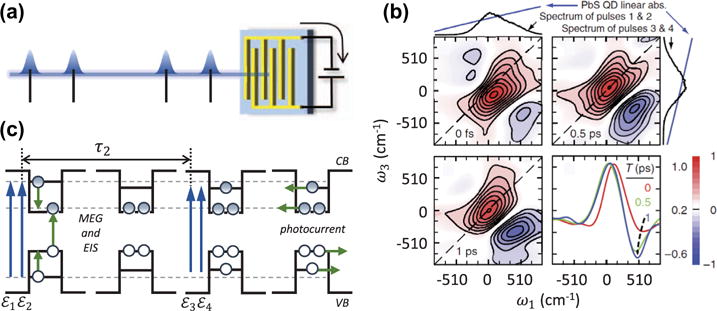
2D photocurrent spectroscopy of a PbS quantum dot photocell at room temperature under vacuum. (a) Collinear pulse sequence and photocurrent detection scheme of the fourth-order population. (b) Real part of the rephasing one-quantum spectrum for various delays τ_2_ up to 1 ps using 351 nm (28,490 cm^−1^ = 3.5 eV = 3.2*E_g_*) excitation. The axes are rescaled relative to the excitation spectrum peak energy. The appearance of a negative feature corresponds to excitation-induced shift (EIS) arising from multi-exciton generation (MEG) after excitation from the first two pulses. The bottom-right panel shows anti-diagonal cross-sections of the 2D spectra, revealing the formation of a dispersive lineshape indicative of MEG as depicted in the energy diagrams in (c).

MDCS with photo-current detection is especially useful when examining photovoltaic materials and other photo-devices, since it provides direct access to ultrafast dynamics of the device under typical operating conditions [147]. Because only current-generating processes can contribute to the detected signal, many background responses such as scattering of the excitation pulses or coherent artifacts are suppressed from detection, resulting in exceptional noise suppression and dynamic range. An elegant example of photo-current MDCS is a recent study investigating carrier multiplication processes in colloidal QD solar cells (see Figure 13(a)) [23]. In these experiments, the sum of the 2D rephasing and non-rephasing spectra (the so-called correlation spectrum) detected through the photocurrent was compared to a similar spectrum measured through fluorescence, both obtained using 351 nm excitation (28,490 cm^−1^ = 3.5 eV). In the photocurrent spectrum, the peak evolved from an absorptive to dispersive lineshape on a picosecond timescale, as shown in Figure 13(b), whereas the lineshape remained primarily absorptive in the fluorescence spectrum. This difference was attributed to multi-exciton generation (MEG) that is only observable in the photocurrent. The interaction of the first two pulses generates a population of excitons with energy equal to ~3.2*E_g_*, where *E_g_* is the bandgap. Non-radiative population relaxation to the band-edge occurs through transfer of energy to excite additional excitons across the bandgap (MEG in Figure 13(c)). Interaction of the final two pulses generates a fourth-order population that is detected through the photocurrent. The many-body interactions between the exciton populations give rise to a dynamic EIS of the excited state that appears within τ_2_ ≈ 1 ps. For similar experiments using 700 nm excitation (~1.6 *E_g_*) EIS is not observed, which is consistent with the absence of MEG for excitation less than 3*E_g_* in PbS QDs [148]. It was speculated that the signatures of MEG were not observed in the fluorescence spectrum for any excitation wavelength due to non-radiative Auger recombination on a timescale faster than the luminescence lifetime but slower than charge carrier separation and photocurrent generation. These results demonstrate how detecting different ‘action variables’ (fluorescence versus photocurrent for these experiments) can provide different physical insight into the mechanisms governing ultrafast carrier dynamics in semiconductor nanostructures.

## 8. Outlook

Multi-dimensional coherent spectroscopy is a powerful tool for probing the structure, composition, and dynamics of condensed matter, atoms, and molecules. The field continues to evolve with new developments in the implementation of the technique and the materials that are accessible. The frequency range of two-dimensional coherent spectroscopy covers terahertz to XUV frequencies. Rapid advances in table-top high-harmonic and X-ray sources have extended this range to enable XUV and X-ray optical spectroscopy of super-excited states and coupled electron-nuclear dynamics in molecules [149,150]. When applied to nanostructures, multi-dimensional XUV/X-ray spectroscopy may improve our understanding of the relation between local atomic structure and the physical properties of low-dimensional semiconductors on a sub-nanometer level and attosecond timescale [151]. These studies may lead to improvements in the realization of nanostructures with optimized size control, tailored structure and material composition, and increased homogeneity and synthesis reproducibility.

Another interesting future direction is to implement multi-dimensional coherent spectroscopy with non-classical light. For example, the unique spectral and temporal characteristics of entangled photons may enhance the experiment sensitivity at lower light levels, improve the selectivity of quantum pathways, and provide better time-and-frequency resolution compared to classical light [152–155]. These advantages could be exploited for controlling exciton transport in nanostructures relevant for energy and charge transfer, such as single-walled carbon nanotubes, quantum dots, and light-harvesting materials.
